# Breeding from 1891 to 2010 did not increase the content of amylase/trypsin-inhibitors in wheat (*Triticum aestivum*)

**DOI:** 10.1038/s41538-023-00219-w

**Published:** 2023-08-23

**Authors:** Sabrina Geisslitz, Darina Pronin, Manjusha Neerukonda, Valentina Curella, Sibylle Neufang, Sandra Koch, Heiko Weichert, Hans Weber, Andreas Börner, Detlef Schuppan, Katharina Anne Scherf

**Affiliations:** 1 Department of Bioactive and Functional Food Chemistry, Institute of Applied Biosciences, Karlsruhe Institute of Technology (KIT), Adenauerring 20 a, 76131 Karlsruhe, Germany; 2 Leibniz-Institute for Food Systems Biology at the Technical University of Munich, Lise-Meitner-Str. 34, 85354 Freising, Germany; 3 Institute of Translational Immunology and Research Center for Immune Therapy, University Medical Center, Langenbeckstr. 1, 55131 Mainz, Germany; 4 Department of Molecular Genetics, Leibniz Institute of Plant Genetics and Crop Plant Research, Corrensstr. 3, 06466 Seeland/OT Gatersleben, Germany; 5 Genebank Department, Leibniz Institute of Plant Genetics and Crop Plant Research, Corrensstr. 3, 06466 Seeland/OT Gatersleben, Germany; 6 Division of Gastroenterology, Beth Israel Deaconess Medical Center, Harvard Medical School, 330 Brookline Ave, Boston, MA 02215 USA

**Keywords:** Plant sciences, Proteins, Proteomics

## Abstract

The prevalence of hypersensitivities towards wheat has increased in the last decades. Apart from celiac disease these include allergic and other inflammatory reactions summarized under the term non-celiac wheat sensitivity. One suspected trigger is the family of amylase/trypsin-inhibitors (ATIs), non-gluten proteins that are prominent wheat allergens and that activate the toll-like receptor 4 on intestinal immune cells to promote intestinal and extra-intestinal inflammation. We therefore quantified 13 ATIs in 60 German hexaploid winter wheat cultivars originating from 1891 to 2010 and harvested in three years by targeted liquid chromatography-tandem mass spectrometry combined with stable isotope dilution assay using specific marker peptides as internal standards. The total ATI content and that of the two major ATIs 0.19 and CM3 did not change from old cultivars (first registered from 1891 to 1950) to modern cultivars (1951–2010). There were also no significant changes in ATI distribution.

## Introduction

Since the first domestication of crops about 10,000 years ago, humans have sought to improve yield, resistance and end-use quality by selecting and breeding cultivars with beneficial traits. For one of the most important crops, wheat (*Triticum aestivum*), the combination of intensified agriculture and new cultivars with high productivity by introducing genes that decrease plant height has resulted in an average grain yield increase by 175% since 1961^1^. In parallel to the improvement of specific wheat traits, such as grain yield, nutrient composition and baking quality, the prevalence of wheat-related disorders, due to consumption, skin contact or inhalation, increased in the last decades^2^. While the reasons for this development are debated, the selection and breeding process of wheat might have led to higher concentrations of constituents that cause symptoms of these disorders.

Wheat-related disorders are generally triggered by wheat proteins^3–5^, which are grouped into water- and salt-soluble albumins and globulins (ALGL) and insoluble storage proteins, the gluten proteins. Gluten proteins are divided into gliadins and glutenins which trigger wheat-related disorders, especially celiac disease in genetically predisposed individuals, and which also contribute to classical (immediate type, immunoglobulin E mediated) wheat allergy and possibly non-celiac wheat sensitivity (NCWS). The term NCWS is used for individuals who report intestinal or extra-intestinal complaints, other than related to celiac disease or classical wheat allergy^2,6^. Apart from gluten proteins, amylase/trypsin-inhibitors (ATIs) are known allergens in bakers’ asthma^7^ and may play a role in wheat-dependent exercise-induced anaphylaxis^8^. Moreover, ATIs activate the toll-like receptor 4 TLR4-MD2-CD14 complex on intestinal monocytes-macrophages and dendritic cells causing their activation and secretion of proinflammatory chemokines and cytokines^9,10^. The activation of these innate immune cells by ATIs is dose-dependent and can exacerbate intestinal and extra-intestinal symptoms typical for NCWS, including the worsening of other pre-existing (autoimmune) diseases in mouse models^11^. Similarly, human studies suggest a role of (non-gluten) wheat proteins, likely also ATIs in exacerbating chronic diseases^12–14^. Because commercially available gluten and gluten-containing food contain ATIs, the exclusion of gluten as trigger of NCWS is difficult^15,16^.

ATIs account for 2.5–6.3% (mean: 4%) of all proteins in wheat^17,18^. Proteins with amylase and/or trypsin inhibitory activity are mapped on 38 genes and proteins with chymotrypsin inhibitory activity on ten genes in the annotated reference genome of wheat cultivar “Chinese Spring”^4,19^. Thirteen ATIs have evidence at protein level in hexaploid common wheat: ATI 0.19, ATI 0.28, ATI 0.53, CM1, CM2, CM3, CM16, CM17, CMX1/3, CMX2, wheat amylase subtilisin inhibitor (WASI), Bowman-Birk type trypsin inhibitor (WTI) and wheat chymotrypsin inhibitor (WCI). The ATIs 0.19 (0.9 mg/g on average in wheat flour from different genotypes and locations) and CM3 (0.7 mg/g) were the two most abundant ones^18^. This is also important, since both recombinant ATIs 0.19 and CM3 were shown to activate TLR4 in vitro and in vivo^10^. ATIs are resistant to digestive enzymes of insects and mammals because of their compact three-dimensional structure, resulting from four to five intramolecular disulfide bonds^20,21^. Disulfide bond reduction and unfolding abolishes their TLR4 activity and makes them susceptible to protease-mediated digestion^9^.

The concentration of ATIs can vary widely between different wheat cultivars and even when a defined cultivar is grown at different sites or in different years^22–26^. First studies indicate that older cultivars (1921–1960) have a similar mean ATI content as modern wheat cultivars (1961–2013)^24^. The genetic control appears to be lower than for, e.g., gluten proteins. Still, it was found to be high for Australian cultivars^22^, but low for European cultivars^24^, where this was attributed to a complex genetic architecture. Therefore, different conclusions were drawn whether targeted wheat breeding can reliably reduce ATI content. Alternatively, genome editing by Crispr-Cas9 or RNAi mediated gene silencing has been used to reduce ATI expression^27,28^.

Breeding of new wheat cultivars has decreased the ratio of gliadins to glutenins from 1891 to 2010^23,29^. Moreover, plant height decreased by about 40%, while yield doubled on average during the same time period^1,29^.

Along with the changes in agronomic characteristics and protein composition, we hypothesize that the rise of wheat-related disorders might be associated with an increasing content of ATIs in modern versus old wheat cultivars. The aim of this study was to elucidate if the ATI content increased due to breeding since 1891. To this aim, the ATI content was determined in 60 wheat cultivars each harvested in the years 2015, 2017 and 2019 in Germany, all having been first registered between 1891 and 2010. This allowed to compare the different old and modern cultivars but also to assess the environmental effects in the years 2015, 2017 and 2019 on the ATI composition.

## Results

We analyzed a representative collection comprising 60 wheat cultivars (*Triticum aestivum*), which were grown in 2015, 2017 and 2019 representing three biological replicates under different environmental conditions (Supplementary Table 1). The cultivars were first registered in Germany between 1891 and 2010. The five most important cultivars in terms of production were selected for each decade, summing up to twelve sets of five cultivars until 2010^29^. The data of the three harvest years were averaged for most comparisons to eliminate the effect of environmental variability. The ATI content of all samples is summarized in Supplementary Table 2.

### Content of individual ATIs from 1891 to 2010

**Fig. 1:**
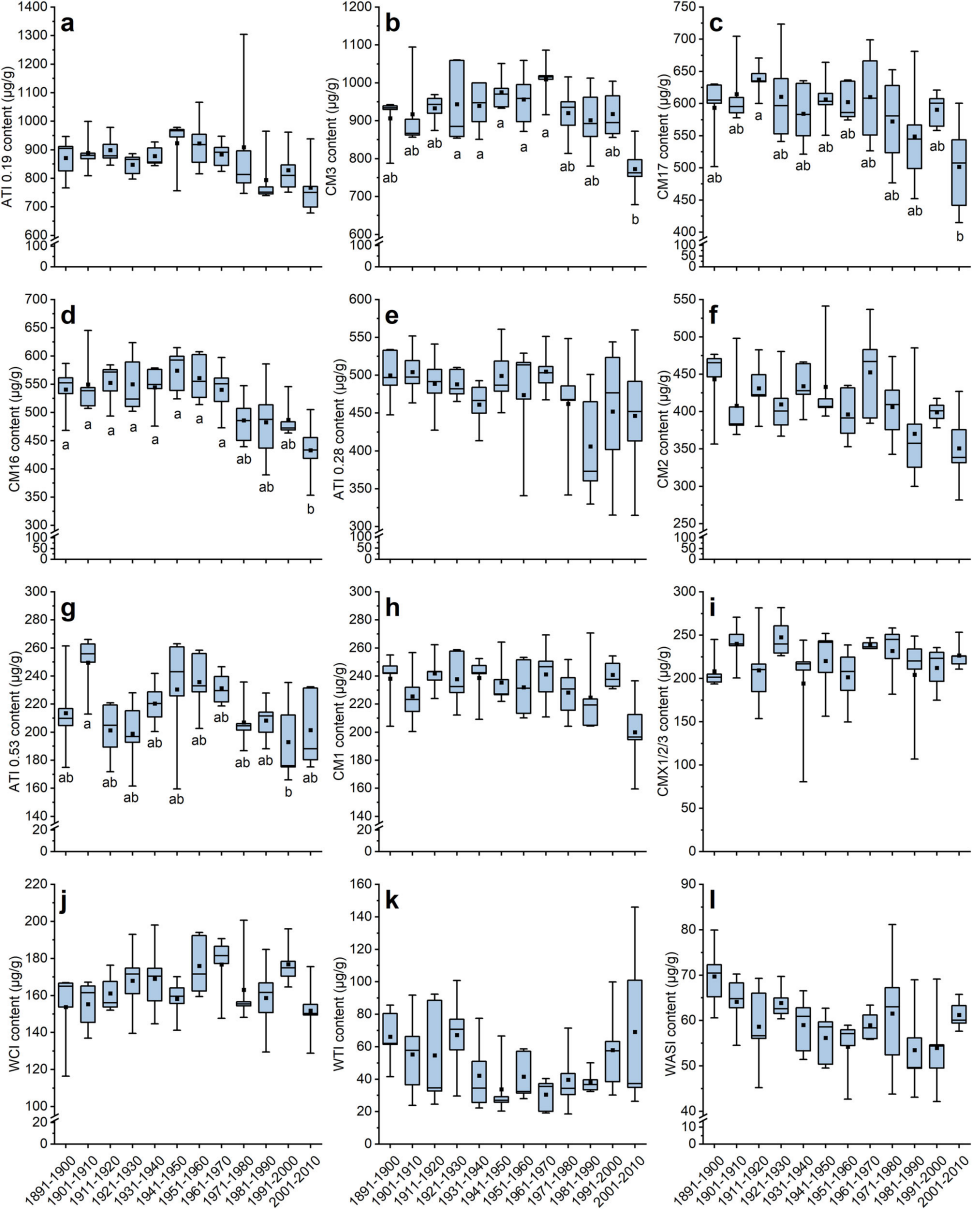
Content of ATIs quantified by LC-MS/MS and SIDA. ATI 0.19 (**a**); ATI CM3 (**b**); ATI CM17 (**c**); CM16 (**d**); CM2 (**e**); 0.28 (**f**); 0.53 (**g**); CM1 (**h**); CMX1/2/3 (**i**); WCI (**j**); WTI (**k**) and WASI (**l**). Mean of five cultivars per decade averaged over three harvest years (2015, 2017 and 2019), respectively. The whiskers refer to the highest and lowest content, the box refers to the interquartile range, the point in the box to the mean and the line to the median. Different small letters indicate significant differences between the decades (one-way ANOVA with Tukey’s test, *p* < 0.05).

Wheat flours were analyzed for their ATI content by SIDA and LC-MS/MS^18^. The two most abundant ATIs were 0.19 (679–1304 µg/g) and CM3 (678–1094 µg/g) (Fig. 1a, b), followed by CM17 (415–724 µg/g), CM16 (354–645 µg/g), CM2 (281–541 µg/g) and 0.28 (314–561 µg/g) (Fig. 1c–f). Minor ATIs were 0.53 (160–266 µg/g), CM1 (160–271 µg/g), CMX1/2/3 (81–282 µg/g), WCI (116–201 µg/g), WTI (19–146 µg/g) and WASI (42–81 µg/g) (Fig. 1g–l). The content of the individual ATIs varied for each decade as is apparent from the size of the boxes and the length of the whiskers in Fig. 1. The relative standard deviation over all 60 samples ranged from 9.3% (CM1) to 53.6% (WTI) and it was also in the same range considering only the five cultivars per decade, respectively. This resulted in no significant differences between the decades for 8 of the 12 ATIs (ANOVA, *p* > 0.05). For three ATIs, the most modern cultivars (2001–2010) had a significantly lower content than those of one old decade (CM17; 1911–1920) or than several decades (CM16; 1891–1970 and CM3; 1920–1970) (ANOVA, *p* ≤ 0.05). For 0.53, there was only a significant difference between the cultivars from 1901 to 1910 and those from 1991 to 2000. Similar trends, even if not significant, were observed for some ATIs (0.19, CM3, CM17, CM16, CM2 and CM1): Old cultivars were characterized by a high ATI content, which either slightly increased or remained constant until 1970 and then slightly decreased resulting in lower ATI content in the modern cultivars.

### Changes of total ATI content from 1891 to 2010

**Fig. 2:**
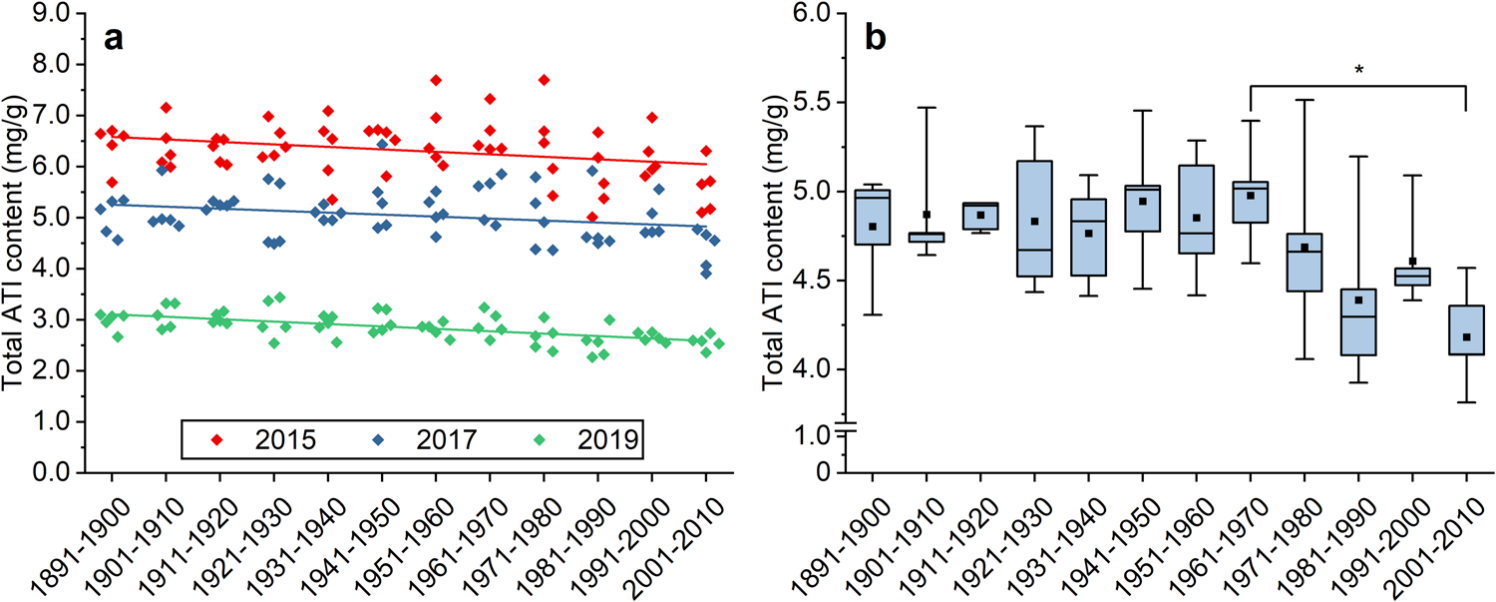
Total ATI content (sum of twelve ATIs) in five cultivars per decade of the harvest years 2015, 2017 and 2019. The line shows a linear regression for each harvest year (2015: *r* = −0.300; 2017: *r* = −0.278; 2019: *r* = −0.613). Exactly the same can be seen for ATI 0.19 and CM3 as most abundant and bioactive ATIs (data not shown) (**a**). Total ATI content (sum of twelve ATIs) in five cultivars per decade averaged over the three harvest years, respectively. The * indicates a significant difference between samples of the two decades (one-way ANOVA with Tukey’s test, *p* < 0.05) (**b**).

The content of all individual ATIs was summed up per cultivar from each harvest year to calculate the total ATI content. The highest total ATI content (mean 6.3 mg/g; 5.0–7.7 mg/g) for the 60 cultivars was observed for 2015, followed by 2017 (mean 5.0 mg/g; 3.9–6.4 mg/g) (Fig. 2a) and 2019 (mean 2.8 mg/g; 2.3–3.4 mg/g). A linear regression for the total ATI content of each cultivar of each harvest year showed a slightly descending line from the oldest to the most modern samples (Fig. 2a). The same decreasing trend was present considering the mean value of each cultivar averaged over the three years (Fig. 2b). Overall, the total ATI content was significantly affected more by the environmental conditions in the three years than by the genetic background of the cultivars (two-way ANOVA: F-value harvest year: 13,653; *F*-value cultivar: 80; *F*-value interaction: 16; *p* < 0.001).

In concordance with individual ATIs, the total ATI content was characterized by a high variation of the five samples within each decade leading to only one significant difference between the decades 1961–1970 and 2001–2010 (Fig. 2b). The trend described above for some individual ATIs was reflected again by an initially constant total ATI content from 1891 to 1970 and then a slightly decreasing trend. The absolute total ATI content varied between 3.8 mg/g and 5.5 mg/g for the average of the three harvest years. The data were distributed normally around the mean (Shapiro-Wilk test, *p* < 0.05) and 44 of 60 cultivars had a total ATI content between 4.4 and 5.2 mg/g.

### Changes in the percentage of total ATI from 1891 to 2010

**Fig. 3:**
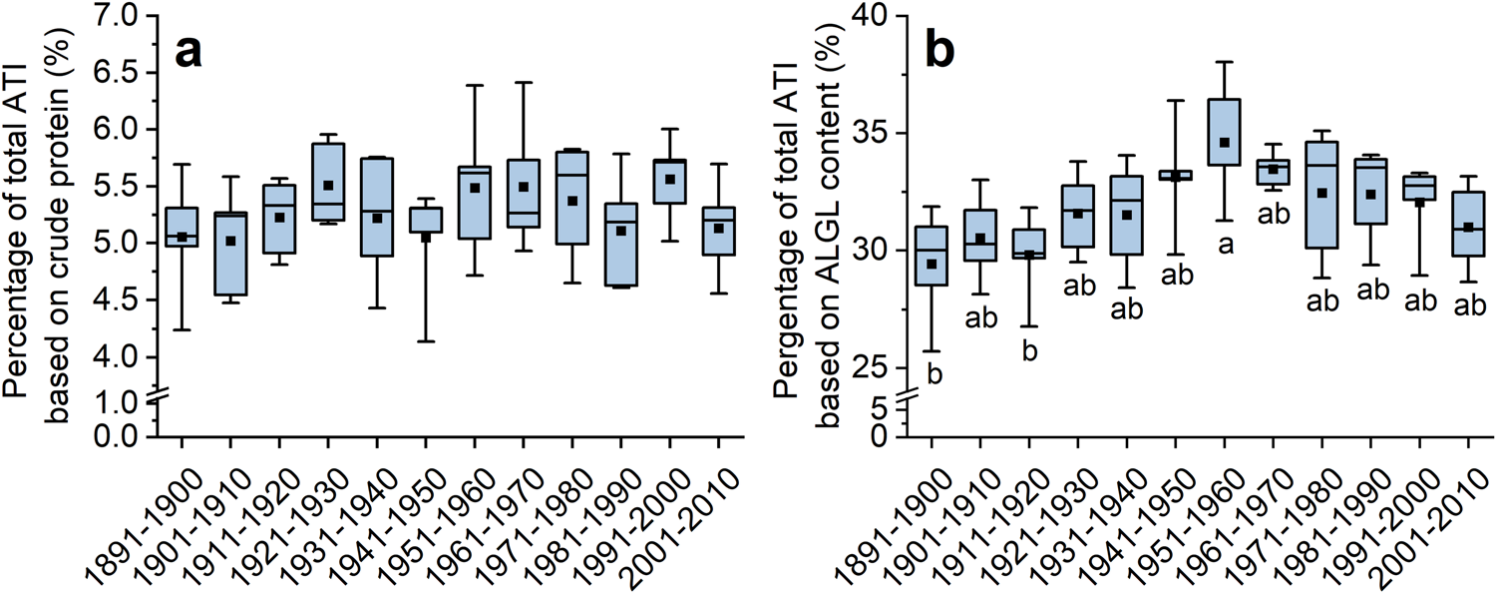
Percentage of total ATI of five cultivars per decade averaged over three harvest years. The percentage is based on crude protein content (**a**) and based on albumin/globulin (ALGL) content (**b**). The whiskers refer to the highest and lowest content, the box refers to the interquartile range, the point in the box to the mean and the line to the median. Different small letters indicate significant differences between the decades (one-way ANOVA with Tukey’s test, *p* < 0.05).

The crude protein content was almost constant for 2017 and showed a decreasing trend from old to modern cultivars for 2015^29^. The samples from 2019 showed a slightly decreasing trend from old to modern cultivars, similar to those from 2015 (Supplementary Fig. 1). The percentage of total ATI based on the crude protein content was 2.4–3.5% for 2019, being lower than in 2015 (4.4–8.0%) and 2017 (4.2–8.2%). In contrast to the absolute total ATI content (Fig. 2b), the percentage of total ATI based on protein was constant for 2017 and 2019 and slightly increased for 2015. The same constant trend was true for the average of the three years with no significant differences between the decades (Fig. 3a). Thus, not only the absolute ATI content, but also the percentage of ATI relative to protein content showed no changes from old to modern wheat cultivars.

ATIs belong to the ALGL fraction which contains proteins that are soluble in water and salt solutions. For 2019, the percentage of ATIs based on ALGL was lower (14.4–30.0%) than for 2015 (28.9–49.5%) and 2017 (28.7–45.4%). For the average of the three years, an increasing trend was observed between 1891 and 1960, followed by a subsequent decreasing trend until 2010 (Fig. 3b). There was no significant difference between old and modern cultivars (one-way ANOVA, *p* > 0.05). As for the absolute total ATI content, there was no increase in the proportion of ATI in ALGL. On average, the ATIs corresponded to about one third of ALGL.

### Changes of ATI distribution from 1891 to 2010

**Fig. 4:**
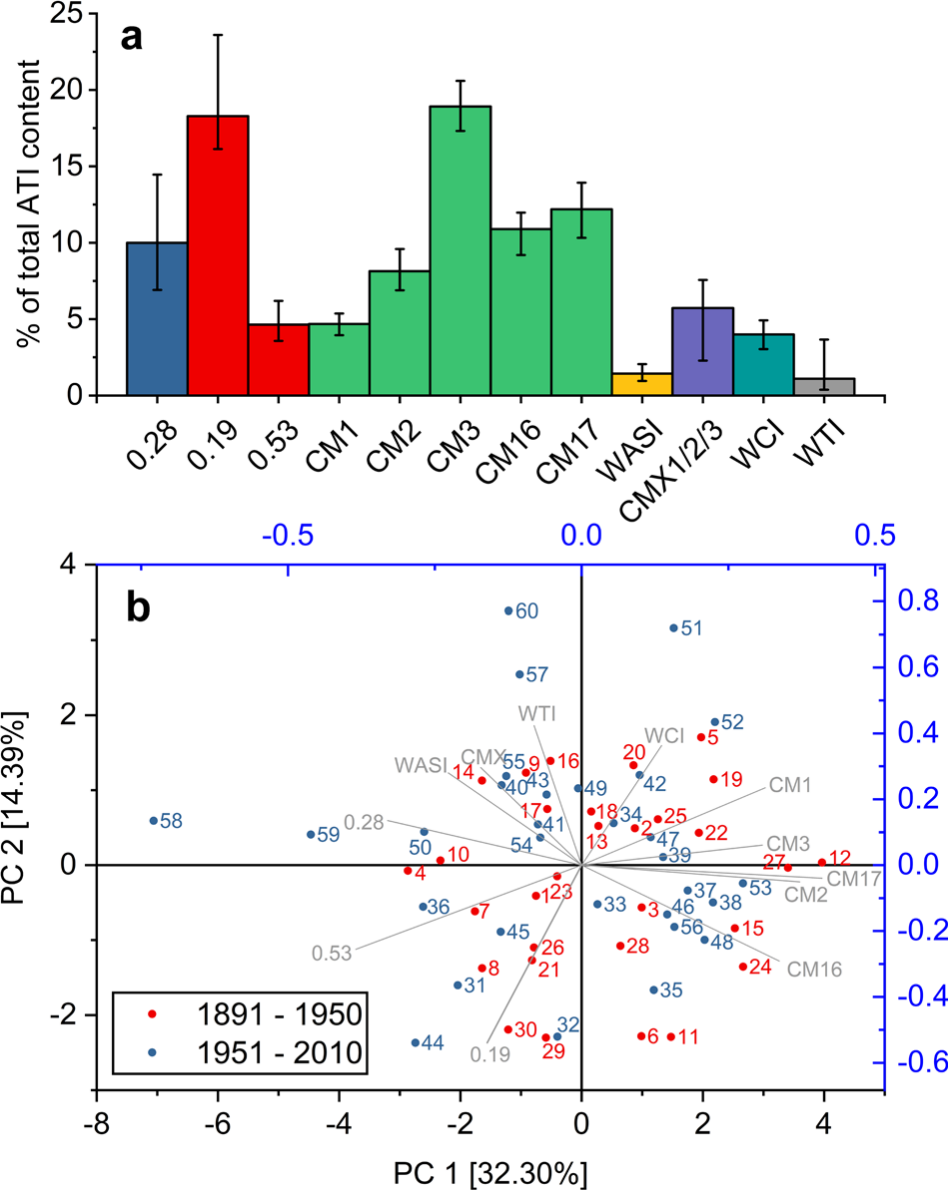
Mean percentage of twelve ATIs in 60 wheat cultivars from 1891 to 2010 from three harvest years (2015, 2017 and 2019). Whiskers represent the respective maximum and minimum. ATIs are displayed as in ref. ^30^ (**a**). Principal component (PC) analysis of relative ATI distribution of old (red, 1891–1950) and modern (blue, 1951–2010) wheat cultivars based on the mean of the three harvest years (**b**).

ATIs 0.19 (16.1–23.6%) and CM3 (17.3–20.6%) corresponded on average to more than one third (37%) of all ATIs (Fig. 4a). ATIs 0.19, 0.28 (6.9–14.5%) and 0.53 (3.6–6.2%) made up 33% on average of all ATIs and they mainly display amylase inhibitory activity (AIs). CM proteins (CM1, CM2, CM3, CM16 and CM17) represented more than one half (55%) of all ATIs, while the remaining 12% were due to the minor ATIs WASI, CMX1/2/3, WCI and WTI. Based on the percentage distribution of all ATIs, a PCA was performed to analyze how far the distribution of individual ATIs changed from old to modern cultivars (Fig. 4b). The first two components accounted for 47% of total variance. The CM proteins and minor ATIs were correlated, respectively, as well as the ATIs 0.19, 0.28 and 0.53. Old and modern cultivars were equally distributed in the score plot with no cluster formation either for old or for modern cultivars. Nevertheless, four cultivars of the most recent decade (2001–2010; 57, 58, 59 and 60) were separated from all other cultivars. However, the fifth cultivar of this decade (56) was located in between old and modern cultivars. A closer look at cultivars 58 and 59 showed a higher ratio between AIs and CM proteins (39/48 and 36/50, respectively) compared to the averaged ratio of old (33/55) and modern cultivars (33/54). However, this higher ratio was not unique for modern cultivars, because one very old cultivar (4) had a comparable ratio (35/52) to the modern cultivars 58 and 59. The difference to 100% corresponded to the minor ATIs. The other two cultivars 57 and 60 were characterized by a high proportion of WTI (2.4% and 3.7%, respectively) that was higher than the average percentage for old (1.2%) and modern (1.0%) cultivars. This high percentage of WTI in 57 and 60 depended on the harvest year. For 2017 and 2019, 57 and 60 had the highest WTI percentage within the complete sample set, but for 2015, sample 51 had the highest WTI percentage (3.4%) followed by 60 (2.1%). Seven samples from decades between 1891 and 1930 had higher proportions of WTI (1.6–1.9%) than sample 57 in 2015.

Overall, the ATI distribution was slightly different depending on the genetic background, but there was no clear change in the ATI distribution from old to modern wheat cultivars, suggesting that there may be some but not a stringent genetic control that balances proportions of the different ATIs.

### Correlation of total ATI and individual ATI content

**Fig. 5:**
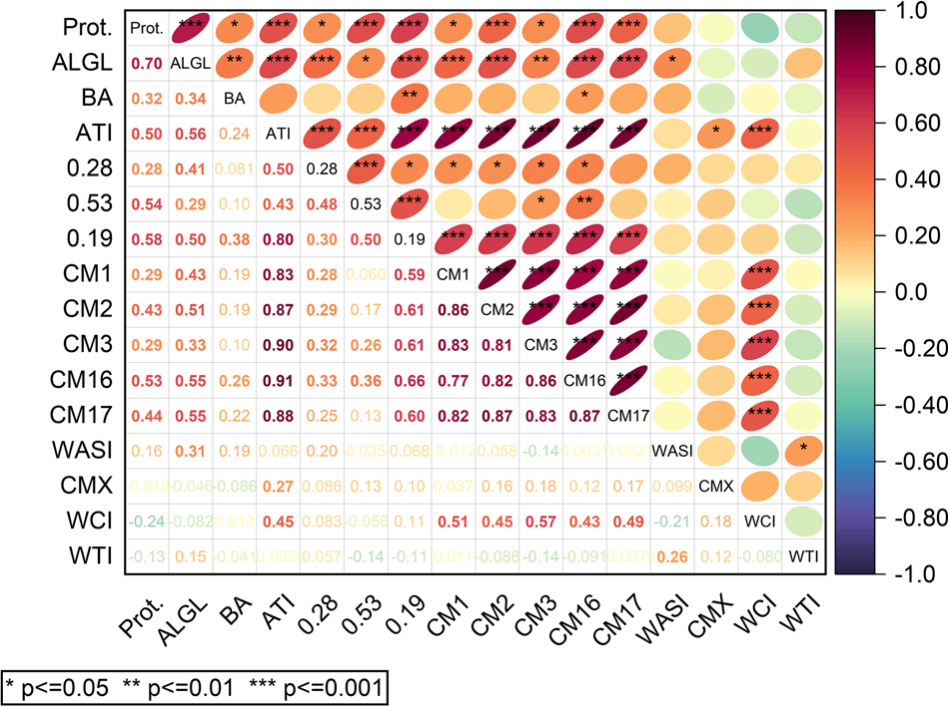
Correlation plot of crude protein content (Prot.), albumin/globulin (ALGL) content, total ATI content (ATI) and individual ATIs. The values are the means of the three harvest years. Significant Pearson correlation values (*p* < 0.05) are bold.

Strong correlations with very high significance (*p* < 0.001) and high Pearson correlation coefficients were observed between the five CM proteins (*r* = 0.77–0.87), between 0.19 and the five CM proteins (*r* = 0.59–0.66), between total ATI content and AIs (*r* = 0.43–0.80) and between total ATI content and CM proteins (*r* = 0.83–0.91) (Fig. 5). These observations were confirmed with at least a significance level of *p* < 0.05 considering the individual harvest years (Supplementary Fig. 2). The three AIs showed weaker correlations among themselves in comparison to the CM-types. Especially 0.19 and 0.28 were only weakly correlated (*r* = 0.39) for 2017 and the mean of all three years (*r* = 0.30) and no correlation was found for 2015 and 2019. In contrast, 0.53 was significantly correlated to 0.19 (*r* = 0.50) and 0.28 (*r* = 0.48). The minor ATIs WASI, CMX1/2/3 and WTI were either not or weakly correlated with the other parameters for both the individual years and the mean over three years. Only WCI was correlated with CM proteins (*r* = 0.43–0.57).

### Influence of environmental conditions and heritability

The variation of ATI contents can be attributed to genetic factors and environmental factors as well as their interaction. Considering the average of the three years, the total ATI content was correlated with the crude protein content (*r* = 0.50) and ALGL (*r* = 0.56) and this was similar in 2017 (*r* = 0.44 and *r* = 0.60, respectively) and in 2019 (*r* = 0.53 and *r* = 0.59, respectively). However, this correlation was lower (*r* = 0.35 and *r* = 0.33, respectively) in 2015 than in 2017 and 2019. Due to the strong environmental effect that influences ATI percentages, it is not possible to estimate total ATI content from protein or ALGL content. The same pronounced environmental effect was observed for the correlation between the crude protein content, ALGL and individual ATIs, respectively.

The heritability (h^2^) describes the share of genetic variance relative to the total variance, which is the sum of genetic and environmental variance for each parameter. Heritability is between 0 and 1 and the higher the heritability, the higher the genetic effect. Heritability was 0.58–0.90 for single ATIs and 0.81 for total ATIs (Supplementary Table 3). This shows that genetic control seems to be comparatively high, despite the overall differences in absolute content depending on the harvest year.

### Identification of cultivars with high and low ATI content

**Fig. 6:**
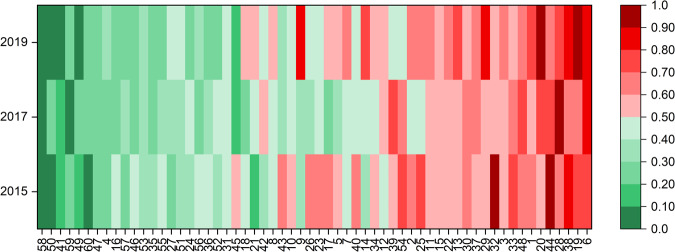
Comparison of the total ATI content of each sample. Values are presented for 2015, 2017 and 2019, respectively. Each column is normalized to values between 0 and 1. Cultivars are sorted by total ATI content from low (left) to high (right).

Considering single cultivars, it was indeed possible to identify cultivars with low or high total ATI content in all three harvest years (Fig. 6). The ATI content of each cultivar from each harvest year (2015, 2017 and 2019) was normalized (0 to 1) to account for the fact that all cultivars generally had a low ATI content in 2019 compared to 2015 and 2017. The cultivar Dekan (58, 2001–2010) was one promising candidate, because the total ATI content was low for all three years. Further promising candidates with low ATI content for at least one year were the modern cultivars Drifter (59) and Tommi (60, both 2001–2010), Okapi (49) and Miras (50, both 1981–1990) and Caribo (41, 1971–1980).

The first 20 cultivars with low ATI content (left of Fig. 6, one third of the sample set) were modern cultivars (1951–2010) with the exception of four older cultivars (4, 16, 24 and 27). Conversely, all 20 cultivars with high ATI content (right of Fig. 6) were older than 1970, with two exceptions (44 and 48). In general, the data in Fig. 6 confirmed the statements made before that the ATI content did not increase due to breeding.

## Discussion

ATI content and composition were first analyzed in the hexaploid wheat cultivar Butte 86 using 2D gel electrophoresis coupled to LC-MS/MS^17,30^. In that study, the percentage of total ATI based on crude protein was 4% compared to 2.4–8.2% of our current set of 180 samples. Overall, the percentage of individual ATIs in Butte 86 was comparable to the mean of all samples in our study (Fig. 4), but there were differences for the pattern of CM-types. Butte 86 had equal proportions of CM2 (12%), CM3 (12%) and CM16 (13%) and lower proportions of CM1 (5%) and CM17 (5%), but we found higher average proportions of CM3 (19%) and CM17 (12%), lower proportions of CM2 (8%) and similar proportions of CM1 (5%) and CM16 (11%) compared to Butte 86. This can easily be explained, since we compared 60 cultivars grown in three years to the single cultivar Butte 86 cultivated in one year, which should lend higher validity as to ATI proportions of our sample set.

The application of SIDA allows the absolute quantitation of peptides and proteins. As alternative, label-free quantitation (LFQ) was used to compare the abundancies of ATIs in wheat^22,25^. The next level instead of using stable isotope labeled peptides are Quantification conCATamers (QconCAT) that consist of artificial proteins designed to release the target peptides during tryptic hydrolysis of the samples. Sielaff et al.^25^ used such ATI QconCAT including 14 peptides to absolutely quantitate eight ATIs (0.19, 0.19-like, 0.28, CM1, CM2, CM3, CM16 and CM17) in hexaploid common wheat and other wheat species. The overall proportion of these ATIs was comparable to that in our current study and our prior study^18^. However, a larger discrepancy was observed for CM3 even though exactly the same peptides were used for quantitation (P9 and P10 in Supplementary Table 4). The most abundant ATI in common wheat was 0.19 (86 nmol/g) using the QconCAT method, but CM3 (26 nmol/g) had equal contents as 0.28 (28 nmol/g), CM16 (29 nmol/g) and CM17 (23 nmol/g). In our study, CM3 (58 nmol/g) and 0.19 (65 nmol/g) showed equal contents, but higher contents compared to CM16 (39 nmol/g) and CM17 (44 nmol/g). The high content of 0.19 and CM3 was confirmed with both analyzed peptides, respectively. Further, using LFQ data we showed already that 0.19 and CM3 were more abundant than 0.28, CM2 and CM16^31^. The observed differences between the studies might either be due to the analysis of different sample sets or to the use of QconCAT instead of stable isotope labeled peptides.

A more cost-effective and less complex setup is the analysis of intact proteins by HPLC-UV. Call et al.^23^ showed that old and modern common wheat cultivars had a comparable ATI content, which is in line with our study. However, using this procedure the relative quantity of individual ATIs cannot be reliably analyzed, since their quantitative separation by HPLC is difficult and fractions are usually contaminated by non-ATI proteins. This is supported by the mostly missing correlation between ALGL and the total ATI content, which we already found in our previous studies^18,31^. This underlines the necessity LC-MS/MS methods using QconCAT or stable isotope labeled peptide standards.

Even if the harvest year had a high influence on the absolute content of individual and total ATIs, the high heritability confirmed that it seems to be possible to select for low ATI content by traditional breeding methods. Moreover, RNAi silencing and CRISPR-Cas9 genome editing already succeeded to silence 0.28, CM3 and CM16 in hexaploid common wheat^28^ and CM3 and CM16 in tetraploid durum wheat^27^, which opens the possibility to eliminate ATIs in a targeted way, a strategy that is currently not approved in the EU.

All plants were harvested together in the last years (2015, 2017 and 2019) including older cultivars that might have had other properties when they were grown in “their” respective decade. Our samples were not fertilized, because we wanted to focus on the effect of genetic changes due to breeding without any additional confounding factor, because it was shown that different time points and ways of fertilization had an influence on the ATI content. Fertilization may be useful to reduce the ATI content^32,33^, but old cultivars cannot be fertilized extensively, because of their tall plant height.

The low total ATI content of the samples harvested in 2019 compared to 2015 and 2017 was noticeable. The different climate conditions in 2019 are the mostly likely reason. The mean precipitation was lower in 2019 (386 mm) than in 2015 (533 mm) and 2017 (557 mm), while the mean temperature in 2019 (10.8 °C) was higher than in 2015 (10.2 °C) and 2017 (10.0 °C). Furthermore, the worldwide atmospheric CO_2_ concentration increased by about 2.5% from 2015 (401 ppm) to 2019 (412 ppm)^34^. However, at present we can only speculate, which of these parameters might indeed have had a major impact on the ATI content. Nevertheless, the hot and dry weather conditions in 2019 might be the most likely determinant, since 2019 was the third warmest year in Germany since 1881^35^. Overall, further research is required to elucidate the effect of precipitation, increasing temperature, rising atmospheric CO_2_ levels and different fertilization treatments on the content of ATIs in common wheat.

There has been much speculation whether breeding during the last decades created modern wheat cultivars with lower nutritional value, lower genetic diversity and higher potential to trigger adverse reactions^36^. Consequently, a lot of research has focused on this controversial statement during the last years. Contrary to the speculations, it has been shown that old and modern cultivars have comparable contents of proteins, lipids, starch and dietary fiber, and therefore do not appear to be different in terms of nutritionally-relevant constituents^37–39^. Overall, our study confirms that old and modern cultivars are comparable in terms of ATI content and composition and this supports previous findings that breeding did not change the immunogenic potential of modern wheat cultivars.

Our hypothesis was that the ATI content might have increased due to wheat breeding and that this may be a reason for the increasing number of patients suffering from wheat-related disorders. In contrast to this, the main conclusion of the current study is that the ATI content did not change from old common wheat cultivars (first registered in Germany from 1891–1950) to modern cultivars (1951–2010). Further, the ATI distribution did not change significantly. Further studies are required to elucidate the correlation between ATI content and their pro-inflammatory effect in autoimmunity and chronic inflammation and inhibitory activity against human amylase^40^. In addition, the effects of food processing including baking, sourdough fermentation and brewing on ATI content and activity are still little understood.

## Methods

### Grain samples

Sixty common wheat (*T. aestivum*) cultivars were selected from the German Federal ex situ Genebank of crops at the Leibniz Institute of Plant Genetics and Crop Plant Research (IPK, Gatersleben, Germany) as already reported in detail by refs. ^29,41^. They represent the five most widely grown cultivars in Germany, which were first registered in the respective decade between 1891 and 2010 (Supplementary Table 1). Cultivars first registered prior to 1951 were designated as “‘old” (samples 1–30) and those registered in 1951 and later as “modern” (samples 31–60).

The samples were cultivated with three biological replicates each at Gatersleben in 2015, 2017 and 2019 and the grains from the three replicates were pooled for each harvest year. The recorded mean temperature was 10.2 °C, 10.0 °C and 10.8 °C for 2015, 2017 and 2019, respectively, and the cumulative precipitation was 533, 557 and 386 mm, respectively (https://wetter.ipk-gatersleben.de/).

The grains from 2015 to 2017 were milled using a laboratory grinder (Bosch, Stuttgart, Germany) and the flours were sieved through a 0.2 mm sieve. Crude protein and ALGL content were already reported by ref. ^29^. The protein content in grains from 2019 was analyzed from intact kernels using a Vario EL Elemental analyzer (Elementar Analysensysteme GmbH, Langenselbold, Germany). The grains from 2019 were milled using an ultra-centrifugal mill ZM 200 (Retsch, Haan, Germany) and the ALGL content was analyzed as described previously^42^.

### LC-MS/MS analysis of ATIs

Sample preparation, stable isotope dilution analysis (SIDA) and LC-MS/MS analysis were performed exactly as reported by ref. ^18^. In short, flour (50 mg) was extracted twice with ammonium bicarbonate (Abic) solution (0.5 mL, 50 mmol/L, pH 7.8) for 30 min at 22 °C. After centrifugation, the combined extracts were evaporated to dryness in a rotational vacuum concentrator (Martin Christ Gefriertrocknungsanlagen GmbH, Osterode, Germany). The residue was dissolved in Tris-HCl (320 µL, 0.5 mol/L, pH 8.5) and 1-propanol (320 µL) and a mixture of internal standards (IS)1–IS20 was added. Reduction was performed with tris(2-carboxyethyl)phosphine for 30 min at 60 °C and alkylation with chloroacetamide for 45 min at 37 °C in the dark. The solvent was removed by evaporation to dryness. Tryptic hydrolysis (0.5 mL, enzyme-to-substrate ratio 1:50, 0.04 mol/L urea in 0.1 mol/L Tris-HCl, pH 7.8) was performed for 18 h overnight at 37 °C in the dark. The reaction was stopped with 2 µL trifluoroacetic acid. The solution was diluted 1 + 1 with 0.5 mL of 0.1% formic acid (FA) and filtered through a 0.45 µm membrane.

An UltiMate 3000 HPLC system (Dionex, Idstein, Germany) was coupled to a triple-stage quadrupole mass spectrometer (TSQ Vantage, ThermoFisher Scientific, Bremen, Germany). An Aqua-C_18_ column (50 mm × 2 mm, 5 µm, 12.5 nm, Phenomenex, Aschaffenburg, Germany) was used with FA (0.1%, v/v) in water as solvent A and FA (0.1%, v/v) in acetonitrile as solvent B. The injection volume was 10 µL, the column temperature 22 °C and the gradient as reported in Geisslitz et al.^18^. Electrospray ionization was performed with source parameters as already reported^18^. Scheduled single reaction monitoring was used to analyze the transitions from precursor to product ions (Supplementary Table 4). Different molar ratios n(peptide)/n(IS) between 9.1 and 0.1 (9 + 1, 4 + 1, 3 + 1, 1 + 1, 1 + 3, 1 + 4 and 1 + 9) were mixed for calibration and analyzed by LC-MS/MS. Data analysis of SIDA was performed as described in ref. ^18^. All detailed results are included in Supplementary Table 2.

### Statistics

PCA, Pearson correlation analysis, one-way ANOVA with Tukey’s test (*p* ≤ 0.05), two-way ANOVA and test for normal distribution (Shapiro-Wilk test, *p* ≤ 0.05) were performed with Origin 2022 (OriginLab, Northampton, MA, USA). Heritability was calculated as *h*^*2*^ = 1 − ϑ/σ^2^_G_, where ϑ is the mean variance of a difference of two best linear unbiased predictors (BLUP) and σ^2^_G_ the genetic variance^43^.

### Reporting summary

Further information on research design is available in the Nature Research Reporting Summary linked to this article.

### Supplementary information

Supplementary Material

Supplementary Table 2

Reporting Summary

## Data Availability

The mass spectrometry data have been deposited to ProteomeXchange Consortium (https://proteomecentral.proteomexchange.org) with the dataset identifier PXD043968 and are publicly available on Panorama Public (https://panoramaweb.org/x9ywP1.url).
