# Supplementary material for: Breeding from 1891 to 2010 did not increase the content of amylase/trypsin-inhibitors in wheat (Triticum aestivum)
Source: NPJ Sci Food. 2023 Aug 23;7:43. doi: 10.1038/s41538-023-00219-w (PMC10447418; doi:10.1038/s41538-023-00219-w)
Supplement: Supplementary file 1 — Supplementary Material [file 41538_2023_219_MOESM1_ESM.pdf]

Breeding from 1891 to 2010 did not increase the content of amylase/trypsin-inhibitors in wheat (*Triticum aestivum*)

Sabrina GEISSLITZ[1,2], Darina PRONIN[2], Manjusha NEERUKONDA[3], Valentina CURELLA[3], Sibylle NEUFANG[3], Sandra KOCH[3], Heiko WEICHERT[4], Hans WEBER[4], Andreas BÖRNER[5], Detlef SCHUPPAN[3,6], Katharina Anne SCHERF[1,2]

[1] Department of Bioactive and Functional Food Chemistry, Institute of Applied Biosciences, Karlsruhe Institute of Technology (KIT), Adenauerring 20 a, 76131 Karlsruhe, Germany

[2] Leibniz-Institute for Food Systems Biology at the Technical University of Munich, Lise-Meitner-Str. 34, 85354 Freising, Germany

[3] Institute of Translational Immunology and Research Center for Immune Therapy, University Medical Center, Langenbeckstr. 1, 55131 Mainz, Germany

[4] Department of Molecular Genetics, Leibniz Institute of Plant Genetics and Crop Plant Research, Corrensstr. 3, 06466 Seeland/OT Gatersleben, Germany

[5] Genebank Department, Leibniz Institute of Plant Genetics and Crop Plant Research, Corrensstr. 3, 06466 Seeland/OT Gatersleben, Germany

[6] Division of Gastroenterology, Beth Israel Deaconess Medical Center, Harvard Medical School, 330 Brookline Ave, Boston, MA 02215, USA

SUPPLEMENTARY MATERIAL

Supplementary Table 1. Overview of the 60 German winter wheat cultivars grown as three biological replicates in three harvest years (2015, 2017 and 2019).

Sample	Decade	Wheat cultivar
1	1891-1900	Rimpaus Früher Bastard
2	1891-1900	Rimpaus Dickkopf
3	1891-1900	Strubes Dickkopf
4	1891-1900	Cimbals Großherzog von Sachsen
5	1891-1900	Steigers Leutewitzer Dickkopf
6	1901-1910	Breustedts Extra Dickkopf
7	1901-1910	Janetzkis Früher Kreuzung
8	1901-1910	Kraffts Siegerländer
9	1901-1910	Ruppiner Brauner Landweizen
10	1901-1910	Ackermanns Brauner Dickkopf
11	1911-1920	Lembkes Obotriten Weizen
12	1911-1920	Heinrichs von Hindenburg
13	1911-1920	Friedrichswerther Berggoldweizen
14	1911-1920	Stadlers Brauner Dickkopf
15	1911-1920	Strubes General von Stöcken
16	1921-1930	Mettes Quedlinburger Schloss
17	1921-1930	Passendorfer Goldweizen
18	1921-1930	Heges Basalt
19	1921-1930	Salzmünder Ella
20	1921-1930	Ackermanns Jubel
21	1931-1940	Heines II
22	1931-1940	Poernbacher Graf Toerring
23	1931-1940	Grundmanns Wotan
24	1931-1940	Nordost Sandomir
25	1931-1940	Rimpaus Bastard II
26	1941-1950	Dippes Strum Weizen
27	1941-1950	Firlbeck I
28	1941-1950	Strengs Marschall
29	1941-1950	Erbachshofer Braun
30	1941-1950	Walz Oberrheinperle
31	1951-1960	Heines VII
32	1951-1960	Carstacht
33	1951-1960	Fanal
34	1951-1960	Merlin
35	1951-1960	Hadmerslebener Qualitas
36	1961-1970	Jubilar
37	1961-1970	Format
38	1961-1970	Konsul
39	1961-1970	Poros
40	1961-1970	Pilot
41	1971-1980	Caribo
42	1971-1980	Diplomat
43	1971-1980	Kormoran
44	1971-1980	Alcedo
45	1971-1980	Vuka
46	1981-1990	Kanzler
47	1981-1990	Ares
48	1981-1990	Rektor
49	1981-1990	Okapi
50	1981-1990	Miras

Supplementary Table 1. Continued.

Sample	Decade	Wheat cultivar
51	1991-2000	Ritmo
52	1991-2000	Zentos
53	1991-2000	Astron
54	1991-2000	Borenos
55	1991-2000	Orestis
56	2001-2010	Akteur
57	2001-2010	Cubus
58	2001-2010	Dekan
59	2001-2010	Drifter
60	2001-2010	Tommi

Supplementary Table 2. The excel file (please see separate file) includes the contents of crude protein, albumins/globulins, total ATI and individual ATIs for the three harvest years 2015, 2017 and 2019. The sum of the quantifier peptides corresponds to the total ATI content. The qualifier peptides were used as confirmation. The table 'Mean_2019-2017-2015' is the respective average of the three harvest years 2015, 2017 and 2019. SD, standard deviation

Supplementary Table 3. Heritability (h^2) of the content of crude protein, albumins/globulins (ALGL), total ATIs and single ATIs based on 60 cultivars and three harvest years.

Compound	Heritability (h^2)
Crude protein	0.57
ALGL	0.70
Total ATI	0.81
0.19	0.74
0.28	0.90
0.53	0.58
CM1	0.67
CM2	0.73
CM3	0.72
CM16	0.77
CM17	0.80
CMX	0.85
WASI	0.88
WCI	0.59
WTI	0.86

Supplementary Table 4. Targeted LC-MS/MS parameters for the quantitation of 13 wheat ATIs. The method has already been published in Geisslitz et al. (2020). P15 was no present in any sample, but was lower than the limit of detection (LOD; peptide level, 1.09 µg/g; protein level, 18.11 µg/g).

Peptide/ internal standard	ATI	Amino acid sequence[1]	Precursor *m/z*		Product ions	Collision energy (eV)	Retention time (min)
			P	IS			
P1/IS1	0.28	LQ**C**VGSQV*PEA*VLR	778.4	784.4	b3/y6/y10/y11/y12	24/23/26/26/25	14.6
P2/IS2	0.28	LTAASVPEV**C***K	587.8	591.8	y2/y5/y7/y8/y9	26/18/17/17/17	11.1
P3/IS3	0.19+0.53	LQ**C**NGSQV*PEA*VLR	785.9	791.9	y4/y6/y7/y8/y10	29/23/25/22/27	13.4
P4/IS4	0.19+0.53	LTAASITAV**C***R	581.8	586.8	y4/y5/y7/y8/y9	16/15/16/16/16	12.6
P5/IS5	0.53	EHGVSEGQAGTGAFPS**C***R	616.3[+]	619.6[+]	b2/b3/y4/y5/y7	29/3021/21/21	10.0
P6/IS6	CM1	SDPNSSVL*K	473.7	477.8	b2/y4/y5/y6/y7	12/17/15/17/12	7.7
P7/IS7	CM2	EYVAQQT**C**GVGIVGSPVSTE*P*GNT*PR	901.8[+]	906.8[+]	b3/y6/y9/y11/y13	36/35/32/34/33	14.2
P8/IS8	CM2	TSDPNSGVL*K	509.3	513.3	b3/y5/y6/y7/y8	13/17/14/14/13	7.6
P9/IS9	CM3	YFIALPVPSQPVDP*R	850.0	855.0	y2/b3/b4/y8/y10	33/27/23/23/23	19.4
P10/IS10	CM3	SGNVGESGLIDL*PG**C***PR	864.4	870.4	y6/y7/y10/y11/y13	29/24/25/25/25	15.9
P11/IS11	CM16	DYVEQQA**C***R	584.8	589.7	b2/y5/y6/y7	19/17/19/21/17	8.3
P12/IS12	CM16	QQ**CC**GE*LANI*PQQ**C**R	931.4	937.9	y5/y6/y7/y8/y9	27/28/26/26/26	11.8
P12c/IS12c	CM16	**Q**Q**CC**GE*LANI*PQQ**C**R	922.9	929.4	y5/y6/y7/y8/y9	27/26/27/27/29	13.3
P13/IS13	CM17	NYVEEQA**C***R	584.8	589.8	b3/y5/y6/y7	19/17/19/21	8.6
P14/IS14	WASI	HVITGPV*R	439.8	444.8	b1/b2/y4/y5/y6	20/15/15/14/13	7.4
P15/IS15	WASI	YSGAEVHEY*K	591.8	595.8	y4/y5/y6/y8/b8	19/18/19/17/18	8.0
P16/IS16	CMX1/2/3	EFIAGIVG*R	481.3	486.3	y3/y4/y5/y6/y7	14/15/15/15/15	15.4
P17/IS17	WCI	ELAAISSN**C***R	560.8	565.8	b3/y5/y6/y7/y8	17/18/18/18/18	9.8
P18/IS18	WCI	AFPPSQSQGGGPPQPPLAP*R	993.5	998.5	y3/y6/y8/y9/y12	38/34/31/33/35	15.5
P19/IS19	WTI	ELEAVSEE**C***R	611.3	616.3	y3/y5/y6/y7/y8	19/1919/19/22	9.6
P20/IS20	WTI	LEGVPEG**C**T*R	559.3	564.3	b2/b4/y5/y6/y8	18/19/25/23/16	9.4

[1] **C**, S-carboxamidomethylcysteine; **Q**, pyroglutamyl; *P, proline ($^{13}C_5$, ^{15}N); *V, valine ($^{13}C_5$, ^{15}N); *K, lysine ($^{13}C_6$, $^{15}N_2$); *R, arginine ($^{13}C_6$, $^{15}N_4$) *G, glycine ($^{13}C_2$, ^{15}N); *L, leucine ($^{13}C_6$, ^{15}N).
[+] precursors were 3^+, all other ones 2^+.

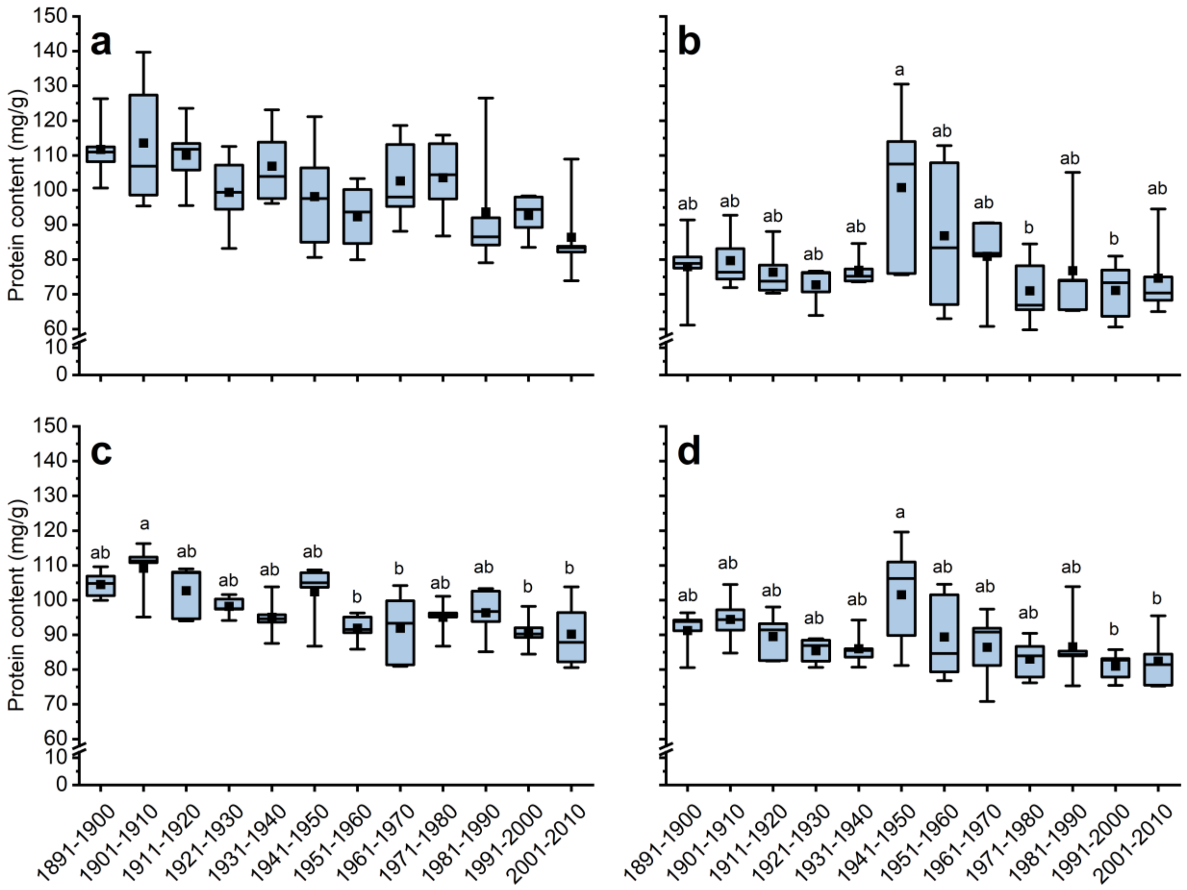

Supplementary Figure 1. Protein content of the samples. a harvest year 2015. **b** harvest year 2017. **c** harvest year 2019. **d** Average of the three harvest years. The whiskers refer to the highest and lowest content, the box to the second and fourth highest content, the point in the box to the mean and the line to the median. Boxes with different lower case letters differ significantly (one-way ANOVA with Tukey's test, $p < 0.05$). The protein content of 2015 and 2017 has already been reported in Pronin et al. (2020) and is displayed to compare those contents with those of the harvest year 2019.

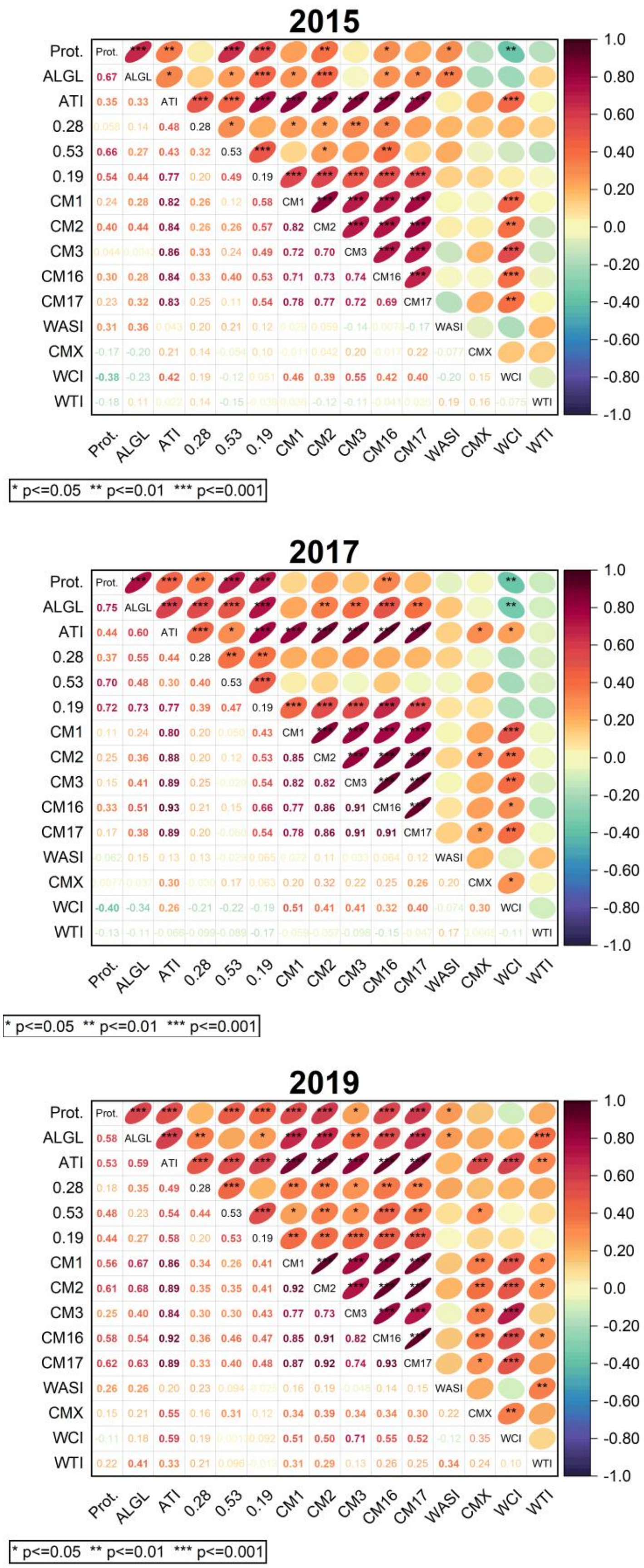

Supplementary Figure 2. Correlation plot of crude protein content (Prot.), albumins and globulins (ALGL), total ATI content (ATI) and individual ATIs (CMX, CMX1/2/3) of the harvest years 2015, 2017 and 2019. Significant Pearson correlation values ($p < 0.05$) are bold.
